# Enhancer of Zeste homolog 2 (EZH2) induces epithelial-mesenchymal transition in endometriosis

**DOI:** 10.1038/s41598-017-06920-7

**Published:** 2017-07-28

**Authors:** Qi Zhang, Peixin Dong, Xishi Liu, Noriaki Sakuragi, Sun-Wei Guo

**Affiliations:** 1 Shanghai OB/GYN Hospital, Fudan University, Shanghai, China; 2 Department of Women’s Health Educational System, Hokkaido University, Sapporo, Japan; 3 Shanghai Key Laboratory of Female Reproductive Endocrine-Related Diseases, Fudan University, Shanghai, China

## Abstract

EZH2, a subunit of the polycomb repressive complex 2 (PRC2) catalyzing trimethylation of histone H3 lysine 27 (H3K27), induces epithelial-mesenchymal transition (EMT) in cancers. However, whether EZH2 regulates EMT in endometriosis is unclear. Here, we show that EZH2 expression, along with its associated PRC2 proteins, is significantly elevated in ectopic and eutopic endometrium from women with endometriosis as compared with control endometrium. EZH2 knockdown or inhibition restored the epithelial phenotypes of endometriotic epithelial cells, concomitant with the upregulation of E-cadherin and downregulation of vimentin and transcription factors (Snail and Slug) as well as reduced cellular migratory and invasive propensity. Conversely, overexpression of EZH2 induced the expression of Snail, Slug and vimentin and suppresses E-cadherin expression. *In vivo* administration of 3-Deazaneplanocin A (DZNep), an EZH2 inhibitor, significantly inhibited the growth of endometriotic lesions and improved generalized hyperalgesia, along with attenuated EMT and reduced fibrosis in endometriosis. Notably, platelets induced EZH2 upregulation and increased H3K27 and H3K9 trimethylation levels in endometriotic epithelial cells. These data identify EZH2 as a novel driver of EMT in endometriosis, implicates the link between wound healing and epigenetic changes in the context of endometriosis, and underscore the role of platelets in the development of endometriosis.

## Introduction

Endometriosis is an estrogen-dependent disorder and a major contributor to pelvic pain and subfertility, affecting 6–10% of women of reproductive age^1^. Characterized by the deposition and growth of functional endometrial-like tissues outside the uterine cavity, it features increased local production of estrogens due to molecular aberrations in steroidogenesis^2^ and is also conceptualized as a pelvic inflammatory condition^3^. Despite exponential growth in the number of publications on endometriosis in the last four decades^4^, its exact pathogenesis and pathophysiology still remain poorly understood^5^.

As noted as early as 1997, one defining feature of endometriotic lesions is their cyclic bleeding as in eutopic endometrium^6^. We have recently demonstrated that, because of this hallmark, endometriotic lesions are essentially wounds that undergo repeated tissue injury and repair (ReTIAR), resulting in platelet-driven epithelial-mesenchymal transition (EMT), fibroblast-to-myofibroblast transdifferentiation (FMT), smooth muscle metaplasia (SMM) and ultimately fibrosis^7, 8^.

EMT is a highly conserved cellular process that allows polarized and generally immotile epithelial cells to convert to motile mesenchymal cells, and occurs in embryonic development, cancer, and wound healing^9^. Not surprisingly, EMT occurs in endometriosis, most likely not because of the increased invasiveness of endometriotic cells *per se* ^10^, but simply because of an innate physiological response to injury due to the nature of lesions undergoing ReTIAR^9^. That is, as wounds undergoing ReTIAR, EMT occurs in endometriosis out of necessity.

Indeed, to date several factors have been shown to regulate EMT in endometriosis and adenomyosis, including estrogen^11^, Wnt/β-catenin^2^, lipocalin 2^12^, hepatocyte growth factor (HGF)^2, 13, 14^, lysyl oxidase^15^, periostin^16^, hypoxia^17^, microRNA-200b (miR-200b)^18^, platelets^7^ and possibly Myc^19^ and LSD1^20^. It becomes clear that EMT not only increases cellular invasiveness but also promotes fibrogenesis^7, 21^.

As in any epithelial cells, the EMT program is orchestrated by multiple signaling pathways as well as epigenetic and post-translational modifications^22^. One potential candidate that is suspected to be involved in epigenetic and post-translational modifications and EMT program in endometriosis is Enhancer of Zeste homolog 2 (EZH2), which was reported to be upregulated in endometriosis^23^.

EZH2 is the catalytic core subunit of Polycomb repressive complex 2 (PRC2), a complex that also includes embryonic ectoderm development (EED) and suppressor of zeste 12 (SUZ12)^24, 25^. PRC2 methylates lysine 27 of histone H3 (H3K27) and lysine 9 of histone H3 (H3K9) to promote transcriptional silencing^24, 25^. EZH2 has emerged as a crucial regulator of wound healing^26^, tumorigenesis^27^, fibrogenesis^28^ and EMT^29, 30^. Mechanistically, EZH2 can directly induce EMT by repressing the expression of E-cadherin through histone H3K27 trimethylation^29^, or indirectly initiate EMT by inhibiting the expression of miR-361 (a suppressor of known EMT inducer Twist)^30^. In addition, several miRNAs have been reported to suppress EMT through EZH2^31, 32^.

Here, we investigated whether EZH2 induces EMT in endometriosis, and whether the specific inhibition of EZH2 could reverse the EMT phenotype, suppress migratory ability of endometriotic cells and retard the growth of endometriosis *in vivo*.

## Results

### EZH2 and other PRC2 constituents are elevated in both ectopic and eutopic endometrium of women with endometriosis

**Figure 1:**
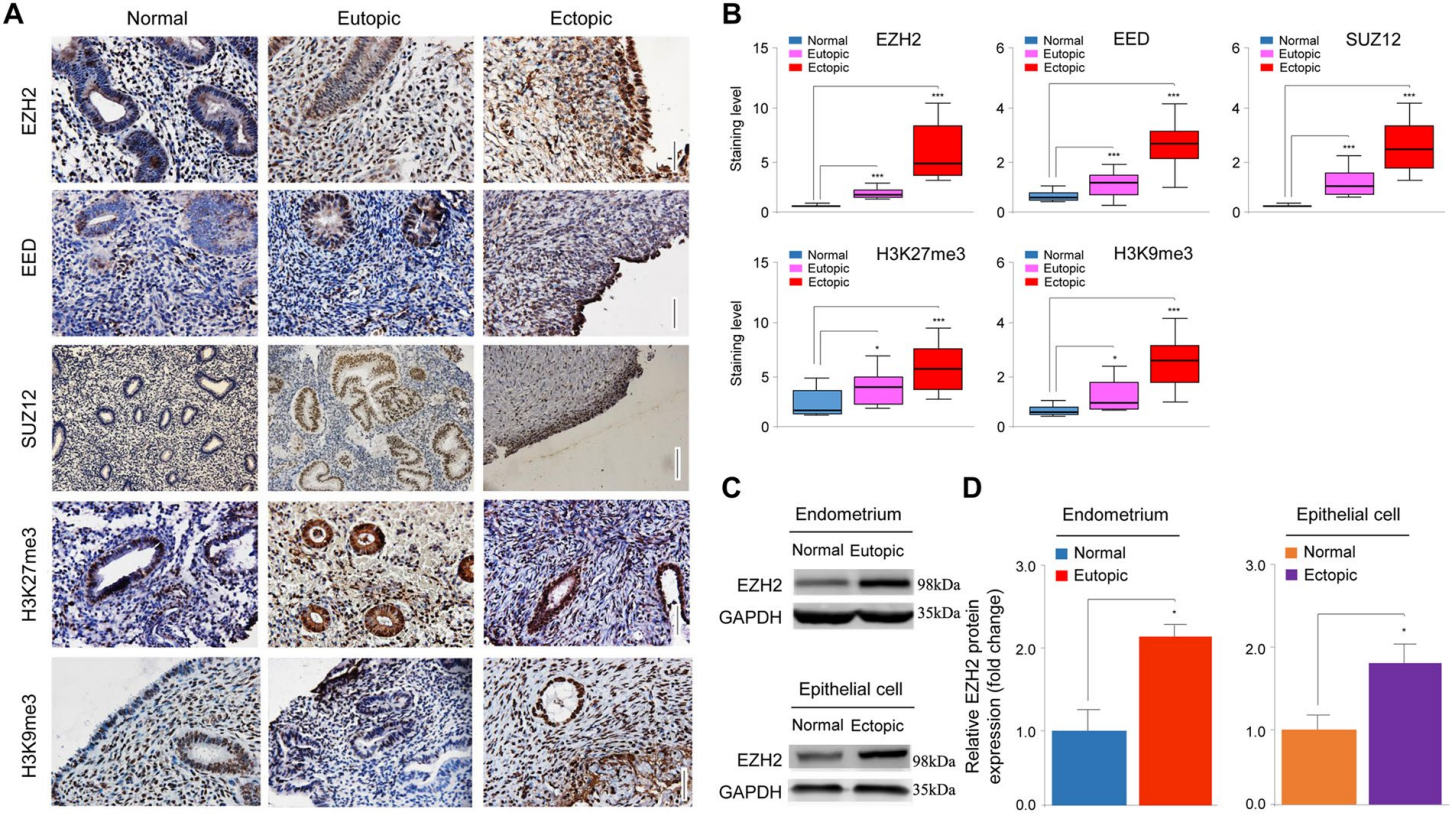
EZH2 expression is elevated in both ectopic and eutopic endometrium of women with endometriosis. (**A**) Immunostaining of normal endometrium, eutopic and ectopic endometrium from patients with endometriosis with antibodies for EZH2, EED, SUZ12, H3K27me3 and H3K9me3 (X400; Scale bar = 125 μm). (**B**) Quantitative comparison of EZH2, EED, SUZ12, H3K27me3 and H3K9me3 protein expression between normal endometrium, eutopic and ectopic endometrium from patients with endometriosis. (**C**,**D**) Representative images (**C**) and quantitative comparison (**D**) of EZH2 protein expression in the whole tissue (including epithelial and stromal cells) between normal and eutopic endometrium (n = 7 each), and in epithelial cells between normal and ectopic endometrium (n = 7 each). The cropped blots are used in the figure. The membranes were cut prior to exposure so that only the portion of gel containing bands in the size range of EZH2 or GAPDH would be visualized, as described in materials and methods. *P < 0.05; ***P < 0.001.

To address the relevance of EZH2 and other PRC2 components to endometriosis, we first performed an immunohistochemistry (IHC) analysis of EZH2, PRC2 constituent proteins (EED and SUZ12), H3K27me3 and H3K9me3 expression in eutopic (n = 14) and ectopic (n = 23) endometrial tissue samples from patients with ovarian endometrioma and in normal endometrial tissues (n = 24). The elevated expression of EZH2, EED, SUZ12, H3K27me3 and H3K9me3 was apparent in ectopic endometrium as compared to eutopic endometrium, especially in the epithelial cells (Fig. 1A). Their protein expression levels were also higher in ectopic and eutopic endometrium as compared with normal endometrium (Fig. 1B). Multiple linear regression controlling for age, parity and menstrual phase yielded identical results (all *p*-values < 0.003, *R* ^*2*^ ranged from 0.38–0.62). There were no statistically significant differences in expression levels of these proteins in the stromal component between eutopic, ectopic and normal endometrium (all *p*-values > 0.05).

We next analyzed the total protein expression of EZH2 by immunoblot in normal and eutopic endometrium, and observed a significant increase in EZH2 expression in eutopic endometrial tissues as compared with normal endometrial tissues (Fig. 1C and D; Supplementary Table S5). In line with this, we found that EZH2 protein expression is significantly up-regulated in epithelial cells derived from ectopic endometrium compared to epithelial cells derived from normal endometrium (Fig. 1C and D; Supplementary Table S5). These data support the hypothesis that EZH2 is involved in endometriosis. In view of the seemingly concomitantly elevated expression of PRC2 constituents and of H3K27me3 and H3K9me3, we conclude that EZH2 is very likely to be functional in the development of endometriosis.

### EZH2 promotes EMT in endometriotic epithelial cells

**Figure 2:**
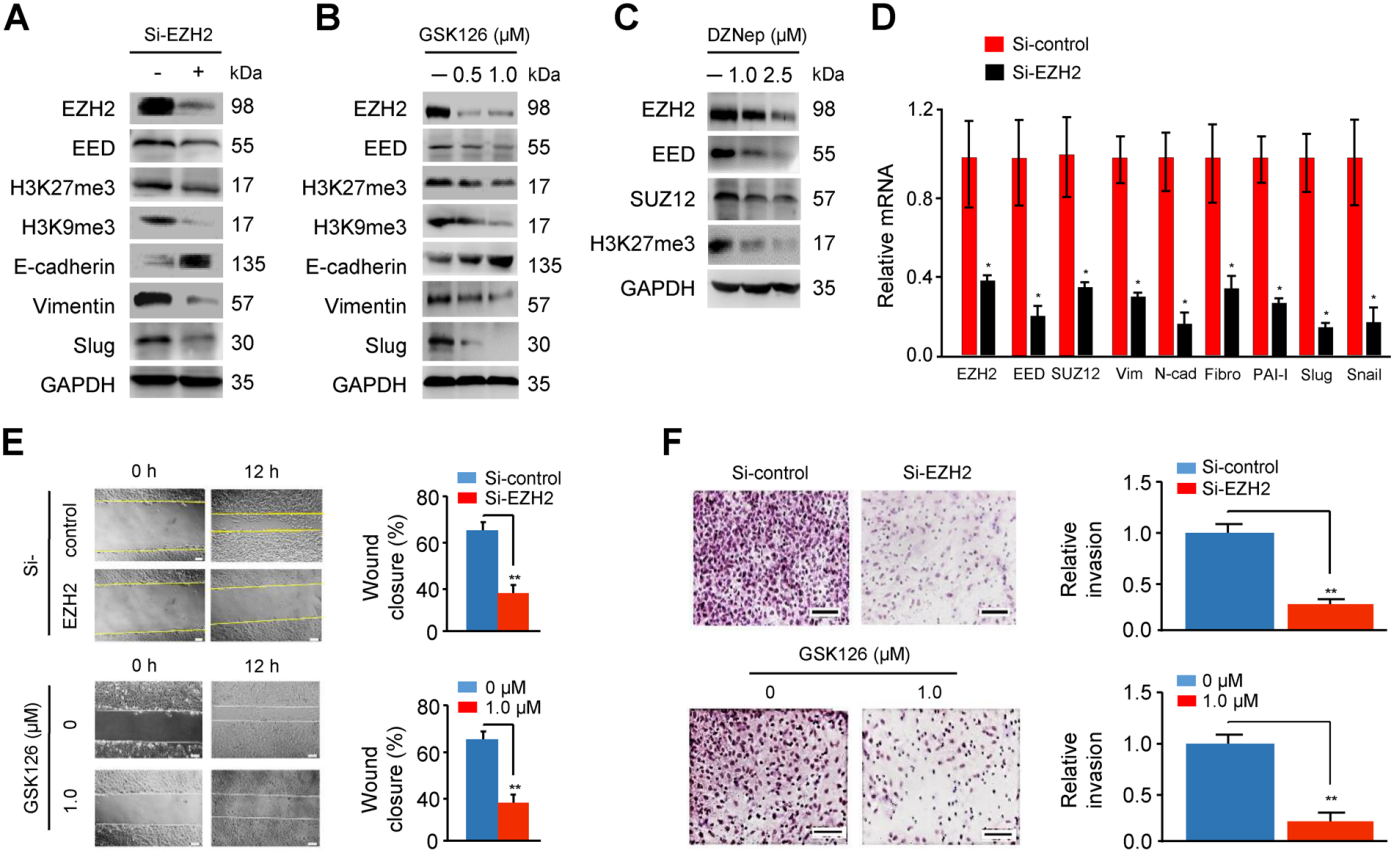
EZH2 promotes EMT in endometriotic epithelial cells. (**A–C**) Western blotting analysis of the indicated proteins in 11Z cells following EZH2 knockdown (n = 5) (**A**), or the treatment with GSK126 (n = 5) (**B**) or DZNep (n = 5) (**C**). The cropped blots are used in the figure. The membranes were cut prior to exposure so that only the portion of gel containing desired bands would be visualized, as described in materials and methods. (**D**) qPCR analysis of the indicated genes in 11Z cells following EZH2 knockdown (n = 5). (**E**,**F**) Migratory (**E**) and invasive (**F**) capacity of 11Z cells following EZH2 knockdown (upper panel) or the treatment with GSK126 (lower panel), was evaluated using wound healing assay and invasion assay (n = 4 each), respectively. Vim, Vimentin; N-cad, N-cadherin; Fibro, Fibronectin. *P < 0.05; **P < 0.01.

To evaluate whether EZH2 knockdown or inhibition could affect the expression of PRC2 protein-coding genes (EED and SUZ12), H3K27me3, H3K9me3 and EMT-related markers, we transiently transfected an endometriotic epithelial cell line 11Z cells with siRNA to knockdown EZH2 expression, or treated 11Z cells with varying concentrations of specific EZH2 inhibitor GSK126 and another pharmacologic EZH2 inhibitor 3-Deazaneplanocin A (DZNep), which inhibits EZH2 expression through effects on intracellular S-adenosyl-L-homocysteine concentrations^33–35^. Both siRNA-mediated EZH2 depletion and the treatment with GSK126/DZNep led to a significant reduction in the trimethylation of H3K27 and H3K9 in 11Z cells, confirming the inhibition of the EZH2 histone methylation activity (Fig. 2A,B and C). In addition, the expression levels of EED, SUZ12, EMT-related transcription factors (Snail and Slug) and mesenchymal markers (vimentin, N-cadherin, fibronectin and PAI-1) were reduced after EZH2 knockdown or the treatment with GSK126/DZNep (Fig. 2A,B,C and D). In contrast, E-cadherin expression was increased following EZH2 knockdown or the treatment with GSK126 (Fig. 2A and B).

**Figure 3:**
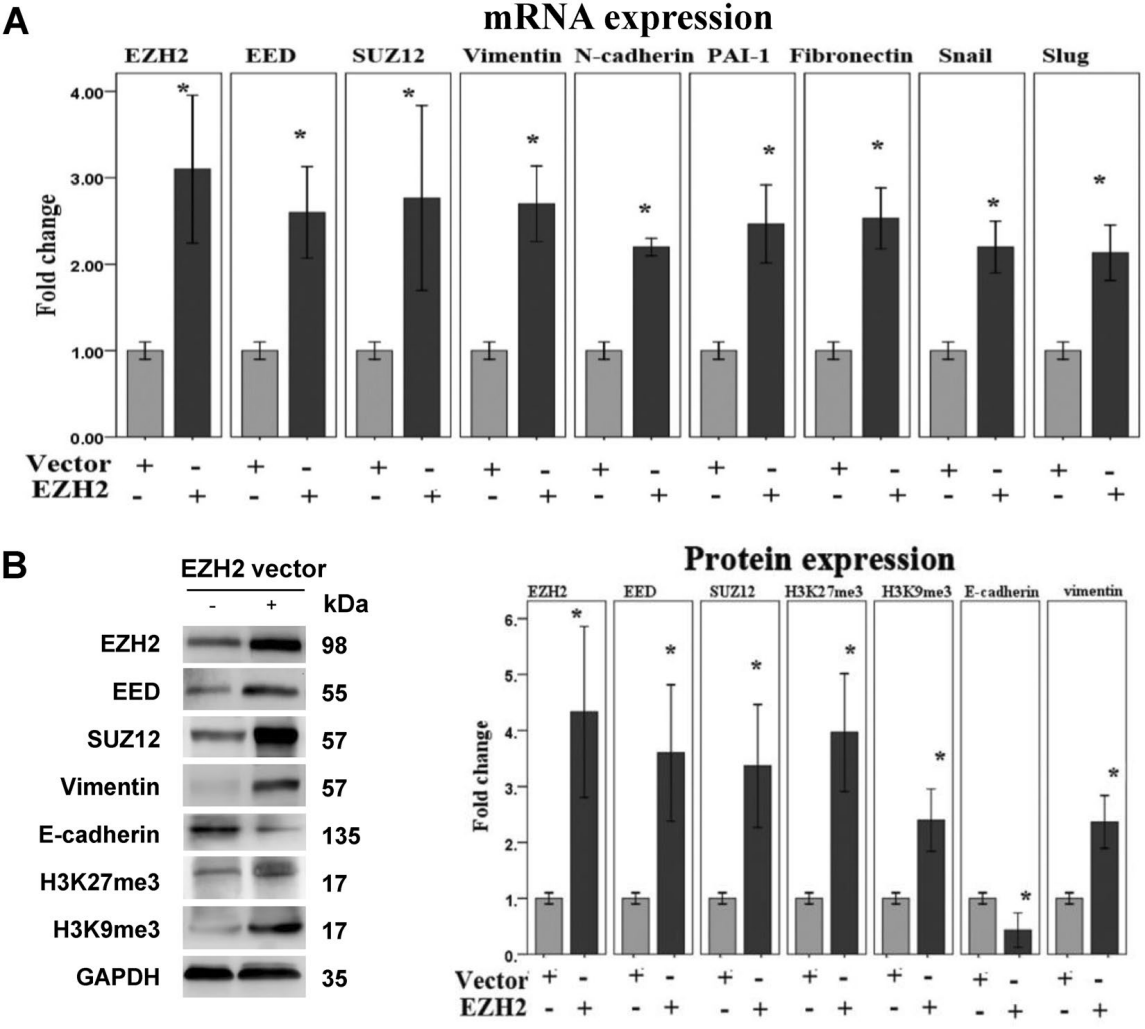
Overexpression of EZH2 promotes EMT in endometriotic epithelial cells. (**A**) qPCR analysis of indicated mRNAs in 11Z cells transfected with empty vector and EZH2 expression plasmids (n = 5). Values are normalized to *GAPDH* expression. (**B**) Representative images (left panel) and quantitative comparison (right panel) of indicated proteins in 11Z cells after overexpression of EZH2 (n = 5). The cropped blots are used in the figure. The membranes were cut prior to exposure so that only the portion of gel containing desired bands would be visualized, as described in materials and methods. ^*^ *P* < 0.05.

Next, we used wound healing assays and transwell invasion assays to examine the effect of EZH2 knockdown or inhibition on 11Z cell migratory and invasive capability. The knockdown of EZH2 or inhibition of EZH2 via GSK126 significantly reduced the migratory and invasive capabilities of 11Z cells (Fig. 2E and F). To further interrogate these findings, we transiently transfected 11Z cells with *EZH2* cDNA plasmid, and tested whether forced EZH2 overexpression could enhance the mesenchymal propensity of this cell line. Indeed, EZH2 overexpression was sufficient to elevate the levels of PRC2 proteins, and induce the expression of H3K27me3, H3K9me3, EMT-inducer genes (Snail and Slug) and various mesenchymal markers while at the same time to reduce the expression of E-cadherin (Fig. 3). Taken together, these data suggest that EZH2 induces EMT to promote migration and invasion of endometriotic epithelial cells.

### EZH2 inhibition by DZNep reduces the growth of endometriosis *in vivo*

**Figure 4:**
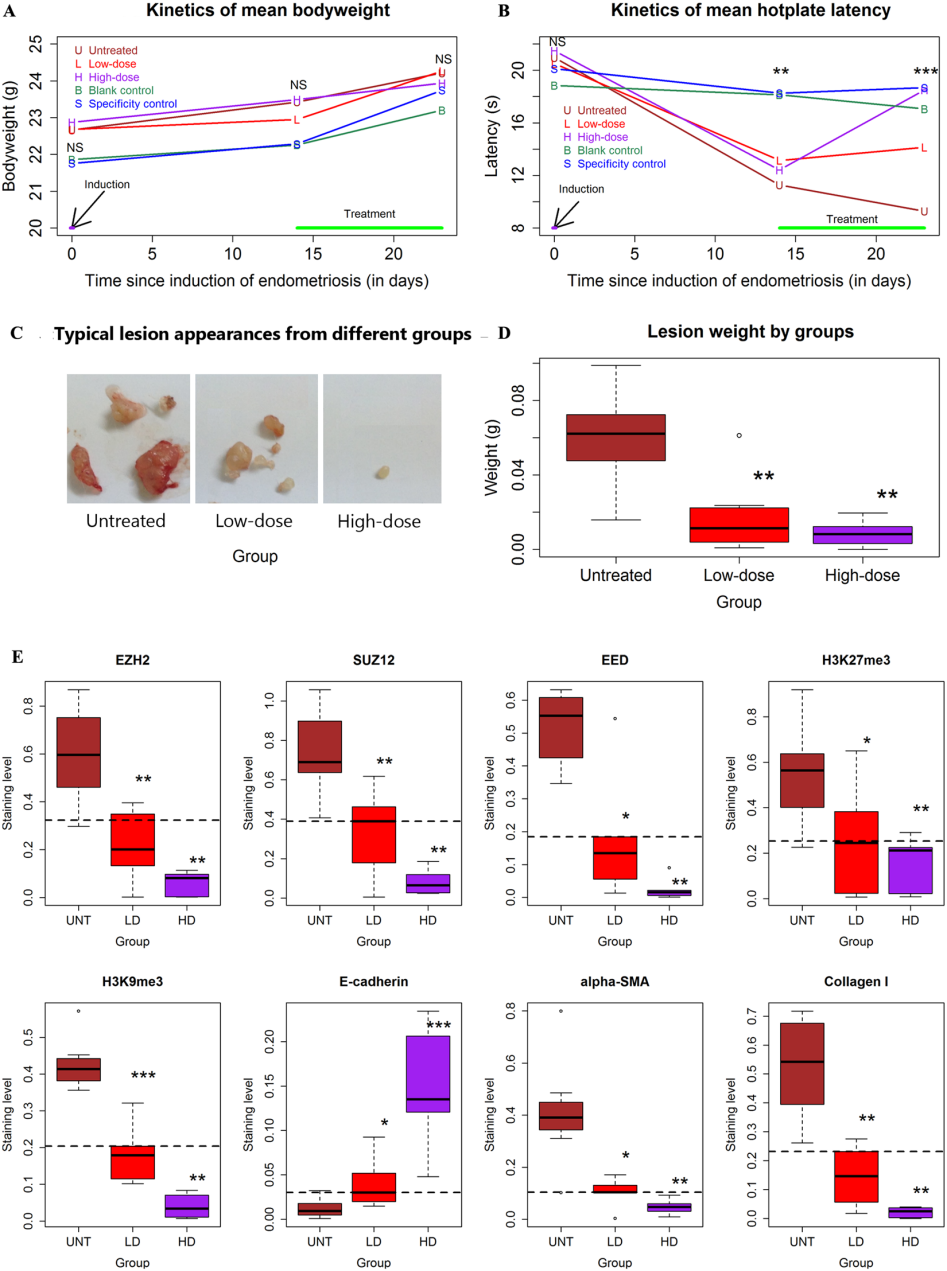
EZH2 inhibition by DZNep reduced the growth of endometriosis *in vivo*, concomitant with suppressed expression of EMT markers and of fibrogenesis. The kinetic changes of bodyweight (**A**) and hotplate latency (**B**) of mice treated with or without DZNep were analyzed. The mice with induced endometriosis were randomly divided into 3 groups of equal sizes: Untreated group (U, n = 8), low-dose DZNep group (L, n = 8) and high-dose DZNep group (H, n = 8). The mice with sham induction were randomly divided into two groups in average: blank control group (B, n = 7) and specificity control group (S, n = 7). (**C** and **D**) Representative images of ectopic lesions (**C**) and quantification of lesion weight (**D**) in mice with induced endometriosis treated with DZNep or vehicle. (**E**) Quantitative comparison of EZH2, EED, SUZ12, H3K27me3, H3K9me3, E-cadherin, α-SMA and Collagen I expression between mice with induced endometriosis treated with or without DZNep. Immunoreactivity against EZH2, EED, SUZ12, H3K27me3, H3K9me3 and E-cadherin in endometriotic epithelial cells was evaluated while the immunoreactivity against α-SMA and collagen I in endometriotic stromal cells was evaluated in mice treated with or without DZNep. DZNep significantly abrogated the expression of EZH2, EED, SUZ12, H3K9me3, H3K27me3, α-SMA and Collagen I while elevated E-cadherin expression in ectopic endometrial epithelial cells, all in a dose-dependent manner. NS: not statistically significant. ^*^ *P* < 0.05; ^**^ *P* < 0.01; ***P < 0.001.

The ability of EZH2 activation to induce EMT in endometriotic epithelial cells raised the possibility that the inhibition of EZH2 by DZNep could suppress EMT and subsequent FMT and fibrogenesis. To test this hypothesis, we conducted a mouse study to see whether EZH2 inhibition using DZNep can suppress EMT and subsequent FMT and fibrogenesis in mice with induced endometriosis. We found that DZNep appeared to be well-tolerated and there was no apparent adverse effect when administered to mice. Neither the induction of endometriosis nor the DZNep treatment had any discernable effect on bodyweight (Fig. 4A and Table S1). As expected, there was no significant difference in hotplate latency prior to the induction of endometriosis (Fig. 4B and Table S2). However, 2 weeks after the induction but before the DZNep treatment, mice with induced endometriosis had a significantly decreased latency as compared with those without (*P = *2.3 × 10^−4^; Fig. 4B and Table S2). Nine days after DZNep treatment, mice with induced endometriosis that received DZNep treatment had significantly longer latency than those without in a dose-dependent manner, contrasting sharply with those untreated mice that experienced deteriorating latency (*P = *0.003; Fig. 4B and Table S2). As expected, the mice in group S and B that had no endometriosis had no significant change in latency (*P = *0.54; Fig. 4B and Table S2), suggesting that the improved latency in mice with endometriosis after the treatment with DZNep, and the improvement was specific due to DZNep treatment.

We found that DZNep significantly suppressed the lesion growth in a dose-dependent manner (*P* = 0.0001; Fig. 4C and D). The mice treated with low-dose DZNep (group L) and with high-dose DZNep (group H) had an average of 71.4% and 86.1% reduction in lesion weight as compared with untreated mice (Fig. 4D).

Our immunohistochemistry analysis of ectopic endometrium suggests that EZH2, EED, SUZ12, H3K27me3 and H3K9me3 staining was seen in cellular nuclei in both stromal and epithelial cells of ectopic lesions but more prominently in epithelium (Supplementary Fig. S1). E-cadherin was expressed mostly in the cytoplasm and membranes of glandular epithelium (Supplementary Fig. S1), and α-SMA and collagen I staining was found in the cytoplasm of stromal cells (Supplementary Fig. S1). We found that DZNep significantly abrogated the expression of EZH2, EED, SUZ12, H3K9me3, H3K27me3, α-SMA and collagen I while elevated E-cadherin expression in ectopic endometrial epithelial cells, all in a dose-dependent manner (all *p*-values < 0.0005 based on linear regression analyses, all *R* ^*2*^ ≥ 0.31; Fig. 4E and Supplementary Fig. S1). DZNep also significantly abrogated the expression of EZH2, EED, SUZ12, H3K9me3 and H3K27me3 in eutopic/control endometrial epithelial cells from mice with or without induced endometriosis, all in a dose-dependent manner (Supplementary Fig. S2). However, there was no significant difference in immunoreactivity against these makers in ectopic and eutopic endometrial stromal cells in different groups. Thus, treatment with DZNep affected expression of the EMT makers in endometriosis.

Of note, the expression of both α-SMA (a marker for myofibroblast) and collagen I (a marker for fibrosis) was significantly reduced by DZNep treatment in a dose-dependent manner in ectopic endometrial stromal cells (Fig. 4E and Supplementary Fig. S1), suggesting the retardation of fibrogenesis as a result of EZH2 inhibition. These data support the notion that EZH2 initiates the EMT (and possibly FMT) in the development of endometriosis, leading ultimately to fibrosis. This provides strong indication that EZH2 may be a potential therapeutic target for the treatment of endometriosis.

### Platelets induce the expression and subsequent activity of EZH2 in 11Z cells

**Figure 5:**
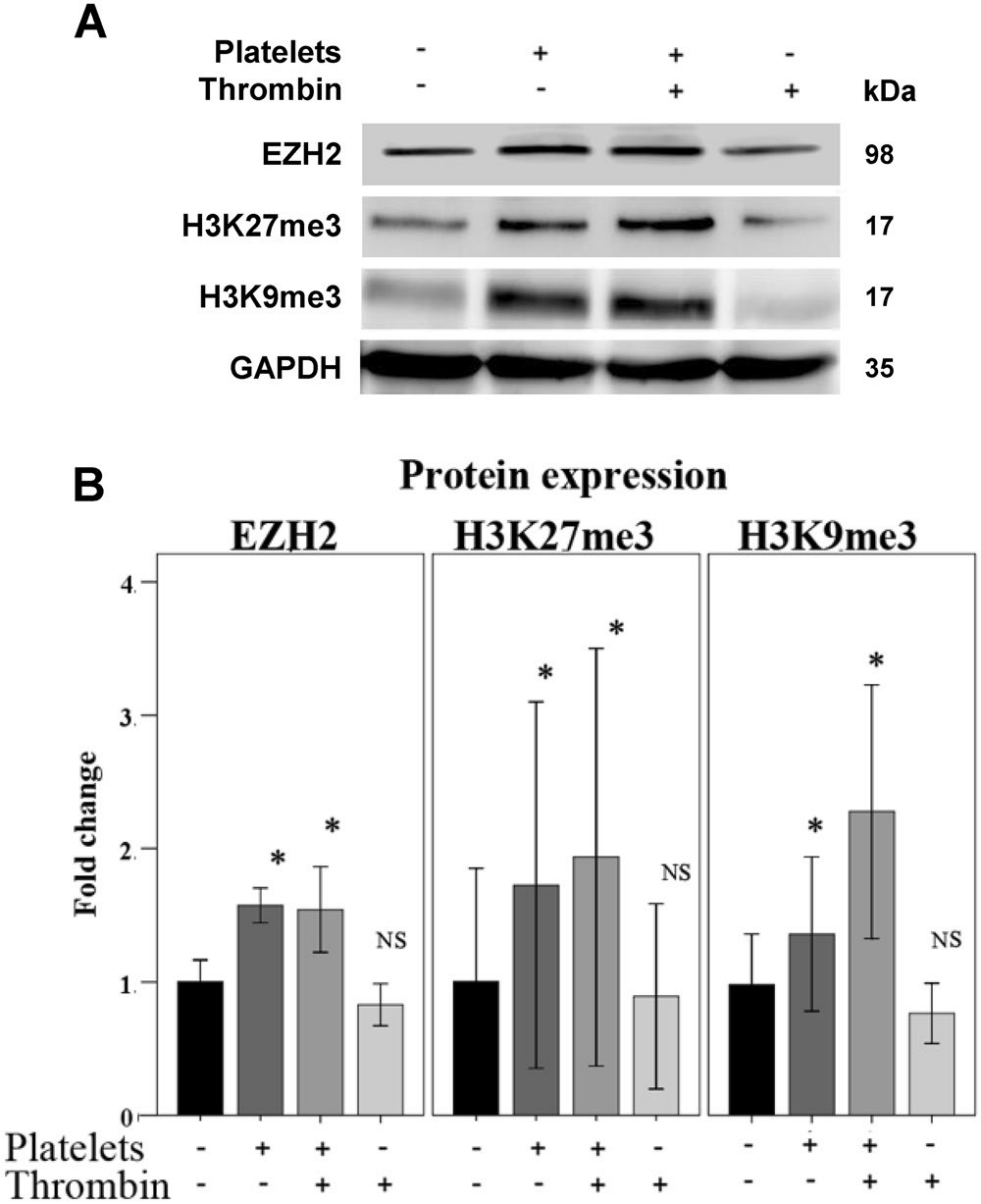
Platelets up-regulate the expression of EZH2, H3K27me3, and H3K9me3 in endometriotic epithelial cells. Western blotting (upper panel) and quantification (lower panel) of EZH2, H3K27me3, and H3K9me3 in 11Z cells treated with buffer (PBS), platelets, activated platelets or thrombin alone (n = 5). NS: not statistically significant. The cropped blots are used in the figure. The membranes were cut prior to exposure so that only the portion of gel containing desired bands would be visualized, as described in materials and methods. ^*^ *P* < 0.05.

We previously reported that platelets induce EMT in endometriotic epithelial cells through activation of the TGF-β/Smad3 signaling pathway^7^. Given the role of EZH2 in inducing EMT as stated above, we asked whether the activated platelets could also induce EZH2 and subsequent H3K27me3 and H3K9me3 expression in endometriotic epithelial cells. After treatment with activated platelets, we observed the upregulation of EZH2 at protein levels and increased protein levels of H3K27me3 and H3K9me3 in 11Z cells (Fig. 5 and Supplementary Table S6), indicating that platelets may promote the EMT properties of endometriotic epithelial cells through up-regulation of EZH2.

## Discussion

In this study, we found that the expression of EZH2 and its associated PRC2 proteins, along with H3K9me3 and H3K27me3, are elevated in endometriosis. Inhibition of EZH2 in endometriotic epithelial cells results in the suppression of the PRC2 protein expression, along with reduced expression of H3K9me3 and H3K27me3 and of key EMT-activating transcription factors. In contrast, forced overexpression of EZH2 results in increased expression of PRC2 proteins and of H3K9me3 and H3K27me3, along with elevated inducers of EMT but reduced E-cadherin expression. Consistently, EZH2 inhibition significantly abolishes migratory and invasive capability of endometriotic epithelial cells. Treatment with an EZH2 inhibitor results in suppression of lesion growth, improved generalized hyperalgesia, and reduced EMT and fibrosis in mouse with induced endometriosis. Finally, we have shown that activated platelets can induce EZH2 expression and its activity in endometriotic epithelial cells.

This study has several strengths. First, we have provided data showing the functions of EZH2, which was previously reported to be over-expressed in endometriosis^23^. Second, we have provided both *in vitro* and *in vivo* evidence to show that inhibition of EZH2 can abolish EMT, thus disrupting the progression of endometriosis development.

This study also has several limitations. First, we did not stain EMT markers in human tissue samples, thus we were not able to show the correlation between PRC2 activation and the extent of EMT. Second, for controls, we used endometrial tissue samples taken from women with benign and potentially malignant diseases but no endometrial abnormality since taking tissue samples from completely disease free-women poses an ethical challenge. While there has been no report that such tissue samples contain molecular or cellular defects that can compromise the results from studies like ours and this seems to be a well accepted practice^36^, the possibility of molecular changes in the endometrium, albeit remote, cannot be completely ruled out. Third, we did not evaluate the effect of platelets on the expression of other PRC2 proteins, such as EED and SUZ12. Hence the precise role of platelets in regulating PRC2 and thus subsequent epigenetic changes still remain to be investigated. Obviously, future research is warranted to illuminate these issues.

Our results are consistent with an earlier report that H3K27me3 levels are elevated in endometriotic lesions (particularly in the epithelium component) and that basal EZH2 expression is elevated in endometriosis^23^. In addition, our findings are in broad agreement with high levels of H3K9me3, H3K27me3 and EZH2 in endometriotic cells^37^ and with increased H3K9 and H3K27 methylation in endometriosis^38^.

One exception is that, while Colon-Caraballo *et al*.^23^ reported that there was no significant difference in the H3K27me3 measured immunostaining levels between ectopic and control endometrium, we found the opposite. This discrepancy could be attributable to the following explanations. First, the 14 endometriotic tissue samples that they used^23^ were from a mixture of ovarian endometrioma (n = 4), superficial peritoneal endometriosis (n = 7), deep infiltrating endometriosis (n = 2) and fallopian tube endometriosis (n = 1). In contrast, all lesion samples in our study came from patients with ovarian endometrioma. As is well accepted, ovarian endometrioma, peritoneal endometriosis and deep infiltrating endometriosis are three separate disease entities^39^. This apparent heterogeneity within the tissue samples^23^ could conceivably reduce the signal-to-noise ratio, obscuring the genuine difference as reported here.

Second, this study had larger sample sizes than that of Colon-Caraballo *et al*.^23^ (the number of ectopic endometrial tissue samples: n = 23 vs. n = 14; number of control endometrial tissue samples: n = 24 vs. n = 15). Everything being equal, studies with smaller sample sizes may not have enough statistical power to detect the difference.

Third, this study evaluated H3K27me3 staining levels exclusively in the epithelial component, which is appropriate due to the EMT. In contrast, Colon-Caraballo *et al*.^23^ did not evaluate the H3K27me3 staining levels separately in different components. In fact, higher H3K27me3 staining levels in the epithelial component than that of the stromal component within the same section are glaring^23^. Again, cell type heterogeneity may also obscure the difference.

Fourth, the same group also reported previously that the methylation levels of H3K27 were significantly higher in endometriotic lesions and endometrium from patients compared to endometrium of controls, using an immunoassay that did not distinguish among mono-, di-, and trimethylation^38^. Our findings, along with increased H3K9me3, are in broad agreement with their results that higher methylation levels of H3K9 were detected in ectopic endometrium^38^.

Lastly, in contrast to tissue array and the immunoassay used by Colon-Caraballo *et al*.^23^, the use of multiple assays, careful choice of cellular component to observe and both *in vivo* and *in vitro* experiments in our study provide pieces of coherent, consistent and biologically plausible evidence that EZH2 and its catalytic product H3K27me3 are aberrantly expressed in endometriosis.

The EMT program can be induced by extrinsic signals such as TGF-β1^40^. Importantly, PRC2 is required for E-cadherin repression^41^ and EZH2 activation can suppress E-cadherin and thus induce EMT^29^. EED, a PRC2 component protein, was shown to regulate TGF-β1-induced EMT in cancer^42^. In fact, in ovarian cancer cells, EZH2 was found to regulate TGF-β1 expression and facilitate invasiveness and metastasis^43^. Thus, EZH2/PRC2 may serve as an important mediator of TGF-β1-induced EMT in endometriosis.

Hypoxia was recently reported to induce EMT in endometriosis^17^. Increased EZH2 expression by hypoxia activates its downstream Wnt/β-catenin pathway in breast cancer^44^. Interestingly, the activation of the Wnt/β-catenin signaling pathway is responsible for EMT induction in endometriosis^21^ and adenomyosis^2^. In addition, hepatocyte growth factor (HGF) can induce EMT in endometriosis^14^ and adenomyosis^13^. Furthermore, EZH2 was shown to silence miR-34a, leading to activation of the HGF/Met/Snail pathway and subsequent EMT in gastric cancer cells^45^, indicating that EZH2 and other PRC2 components might mediate HGF-induced EMT in endometriosis^13, 14, 19^. Since EZH2 can epigenetically silence miR-200b^46^, an EMT suppressor in endometriosis^18^, EZH2 possibly promotes EMT in endometriosis through the inhibition of miR-34a and miR-200b expression. Taken together, EZH2/PRC2 may play a central role in the regulation of EMT process in endometriosis.

Besides its crucial role in EMT induction, EZH2 may also influence development of endometriosis via other mechanisms. EZH2 has a role in the function of natural killer (NK) cells as selective EZH2 depletion or inhibition resulted in increased NK cell activity and NKG2D expression in both murine and human systems^47^. Interestingly, NKG2D expression has been reported to be down-regulated in endometriosis^48^, indicating that EZH2 inhibition may inhibit the progression of endometriosis through mobilization of NK cells. In addition, since endometriosis is now recognized as an epigenetic disease^49^ featuring aberrant DNA methylation^50^ and EZH2 directly controls DNA methylation^24^, EZH2 and its associated PRC2 proteins may thus play important roles in modulating DNA methylation during the progression of endometriosis.

Fibrosis is now recognized as a prominent feature of endometriosis^8, 21^ as well as of adenomyosis^51, 52^. Remarkably, EZH2 is intimately involved in fibrogenesis^28^. As our mouse study indicates, EZH2 inhibition resulted in reduced myofibroblast activation and fibrosis. Consequently, there is a reason to believe that EZH2 plays important roles in the development of endometriosis through facilitating EMT, FMT and fibrogenesis, and causing epigenetic aberrations along the way.

Since activated platelets induce EZH2 expression, it is possible that platelet-induced EMT and fibrogenesis in endometriosis may be mediated, at least in part, by EZH2 and its associated PRC2. Our findings may lead to the development of the anti-platelet/anti-thrombotic therapy for patients with endometriosis^36, 53^.

By showing that EZH2 induces EMT and platelets activates EZH2 expression in endometriotic epithelial cells, this study essentially demonstrates the importance of epigenetic regulation in EMT in endometriosis. More profoundly, it shows that, as wounds undergoing ReTIAR^7, 8^, endometriotic lesions also undergo epigenetic changes as they undergo EMT, FMT, SMM, and fibrogenesis. This, for the first time, implicates the link between wound healing and epigenetic changes in the context of endometriosis.

EZH2 inhibitors have shown promises in cancer clinical trials and become additional strategies to combat cancer^30–35^ and fibrosis^28^. EZH2 inhibitor GSK126 has been used for ovarian and endometrial cancer, lymphoma and melanoma^33, 34^. DZNep, on the other hand, promotes the degradation of the PRC2 complex and indirectly inhibits EZH2 through effects on intracellular S-adenosyl-L-homocysteine concentrations^35^. Our data suggest that EZH2 inhibition via GSK126 and DZNep restored the epithelial phenotypes of endometriotic epithelial cells. It is tempting to propose that EZH2-targeting agents may be useful for endometriosis patients harboring high levels of EZH2.

In summary, the expression of EZH2 and its associated PRC2 proteins, along with H3K9me3 and H3K27me3, are elevated in endometriosis. Inhibition of EZH2 abrogates EMT in endometriotic epithelial cells. Treatment with an EZH2 inhibitor results in suppression of lesion growth and reduced EMT and fibrosis in mouse with induced endometriosis. Activated platelets can induce EZH2 expression, thus activates PRC2 that tri-methylate H3K9 and H3K27. These results piece together numerous findings published so far, that is, EMT, fibrogenesis, and epigenetic aberration, shedding new light onto the molecular mechanisms underlying the development of endometriosis and pointing out new avenues for the development of novel therapeutics.

## Materials and Methods

### Patients and tissue specimens

This study followed the ethical principles outlined by the Helsinki Declaration and was approved by the institutional ethics review board of Shanghai OB/GYN Hospital, Fudan University. After informed consent, paired eutopic and ectopic endometrial tissue samples were obtained from 14 women with endometriosis. In addition, 9 additional ectopic endometrial tissue samples from women with endometriosis were collected. All patients were premenopausal and received no hormonal or anti-coagulant treatment prior to the surgery, and had ovarian endometriomas (rASRM Stage III-IV). The diagnosis of endometriosis was established through laparoscopy and histological confirmation. As controls, endometrial tissue samples were obtained after written informed consent from 24 cycling women, age- and menstrual phase-matched (in frequency) with those with endometriosis, who underwent surgery for teratoma (n = 13, or 52%), cervical intraperitoneal neoplasia (CIN) III (n = 9, or 40%), and tubal ligation (n = 2, or 8%), but free of endometriosis, adenomyosis and endometrial abnormalities, such as uterine fibroids per laparoscopic examination and subsequent histological evaluation. The clinical characteristics of the subjects recruited are shown in Table 1. All subjects were queried about her family history, previous history of deep venous thrombosis and coagulation disorders, and were non-smokers.

**Table 1:** The characteristics of the recruited subjects.

Variable	Control (n = 24)	Endometriosis (n = 23)	*P*-value
Age (in years)	34.0 ± 6.0	31.9 ± 8.1	0.3
Range	31 (25–47)	30 (23–48)	
Menstrual phase			
Proliferative	11 (45.8%)	9 (39.1%)	0.77
Secretory	13 (54.2%)	14 (60.9%)	
Parity			
0	13 (54.2%)	15 (65.2%)	0.76
1	6 (25.0%)	6 (26.1%)	
2	4 (16.7%)	2 (8.7%)	
3	1 (4.2%)	0 (0.0%)	
rASRM stage			
III	—	13 (56.5%)	—
IV		10 (43.5%)	

### Cells and reagents

The endometriotic epithelial cell line 11Z^2^ was kindly provided by Dr. Jung-Hye Choi and was cultured in RPMI-1640 medium (Gibco Laboratories, Grand Island, NY, USA) supplemented with 5% fetal bovine serum (FBS) (Gibco), 100 IU/mL penicillin G, 100 μg/mL streptomycin and 2.5 μg/mL Amphotericin B (Hyclone, Utah, USA). Total cellular proteins were extracted from 11Z cells at 48 h, after the treatment with dimethyl sulfoxide (DMSO) or 3-Deazaneplanocin A (DZNep) (1 or 2.5 μM, Sigma, MO, USA), or the treatment with DMSO or GSK126 (0.5 or 1 μM, Biovision, Mountain View, CA, USA), as previously reported^35, 54^.

### Transient knockdown and overexpression of EZH2 expression and transfection

For transient knockdown of *EZH2*, small interfering RNA (siRNA) against human *EZH2* gene (Si-EZH2, sense: 5′-GGAUGGUACUUUCAUUGAATT-3′ and antisense: 5′-UUCAAUGAAAGUACCAUCCTT-3′) and non-specific control siRNA (Si-control) were purchased from Genepharma (Shanghai, China), and transfected into cells at a final concentration of 30 nM for 48 hours using Lipofectamine 3000 (Invitrogen, Waltham, MA, USA), according to the manufacturer’s instructions. For transient overexpression of *EZH2*, 11Z cells were transfected with an EZH2 expression plasmid (Asia-Vector Biotechnology Co. Ltd., Shanghai, China) for 48 h, using Lipofectamine 3000 (Invitrogen) with an empty plasmid (Asia-vector Biotechnology) serving as control.

### Immunohistochemistry

Human and mice tissues were fixed with 10% neutral formalin and embedded in paraffin for H&E staining and immunohistochemical staining. The paraffin sections were cut to a thickness of 4-μm and were stained with the antibodies against EZH2, EED, SUZ12, H3K27me3, H3K9me3, E-cadherin, α-SMA and collagen I (as shown below). In brief, paraffin sections were subjected to high-temperature antigen retrieval, and stained with the primary antibodies overnight at 4 °C, followed by incubation with HRP-conjugated secondary antibody. The sections were viewed using an Olympus BX53 microscope (Olympus, Tokyo, Japan) and the images were captured using an Olympus DP73 camera (Olympus). Three to five randomly selected fields (400X) of each sample were taken to determine the average optical density using the computer-assisted image system (Image Pro-Plus 6.0, Media Cybernetics, Bethesda, MD, USA). For quantification, the staining levels of EZH2, EED, SUZ12, H3K27me3, H3K9me3, and E-cadherin were evaluated only in control and ectopic endometrial epithelial cells (because of EMT) but that of α-SMA and collagen I was evaluated in the stromal component of respective endometrium (because of fibrosis). The antibodies used in this study are listed in Supplementary Table S3.

### RNA isolation and real-time RT-PCR (qPCR)

Total RNA was extracted from 11Z cells using TRIzol (Invitrogen, Carlsbad, CA, USA), and cDNA was synthesized with the reverse transcription kit (Tiangen, Beijing, China). qPCR was performed using the SYBR Premix Ex Taq (Takara, Tokyo, Japan). *GAPDH* was used for normalization. The primers are listed in Supplementary Table S4.

### Western blotting

Cells were lysed using the Radio-Immunoprecipitation Assay (RIPA) buffer (Fermentas, Waltham, MA, USA). Protein concentrations of the extracts were measured with bicinchoninic acid assay (Beyotime, Shanghai, China). 40 µg cell proteins were applied to 10% SDS-polyacrylamide gel. After electrophoresis, the proteins were transferred to polyvinyl difluoride membranes (Bio-Rad, Hercules, CA, USA). The membranes were incubated at 4 °C overnight with the primary antibodies against EZH2, EED, SUZ12, H3K27me3, H3K9me3, E-cadherin, vimentin, α-SMA, Collagen I or GAPDH. Then the membranes were incubated with HRP-conjugated secondary antibodies for 1 h at room temperature, and finally visualized using enhanced chemiluminescence reagents (Pierce, Waltham, MA, USA). The bands were analyzed by Quantity One software (Bio-Rad), without the use of X-Ray films. The quantitative data were obtained by normalizing to GAPDH signals. The antibodies are listed in Supplementary Table S3.

### Wound healing assay

To examine directional cell migration, *in vitro* wound healing assays were performed as described previously^54^. Briefly, 11Z cells were transfected with EZH2 siRNA (si-EZH2) or treated with GSK126 as described above. Confluent cells were scraped by 100-μl pipette tip to create a wound area, and incubated with fresh medium for 12 h. The 12-h time point was chosen to decrease the potential impact of proliferation on the closing of the scratch. Images of the migration area were captured at 0 and 12 h, and wound healing was quantified using the Image Pro-Plus software 6.0 (Media Cybernetics) as the median percentage of the remaining cell-free area compared to the area of the initial wound.

### Transwell invasion assay

Cell invasion was measured using Matrigel pre-coated transwell inserts (24-well plates; Corning Life Sciences, Tewksbury, MA, USA) as previously described^55^. Briefly, cells (1 × 10^5^) transfected with EZH2 siRNA or treated with GSK126 (1 μM) were seeded in serum-free media onto polycarbonate membrane inserts (8 μm pore size). Inserts were then submerged in media containing 20% FBS, and the cells were incubated for 48 h. Our MTT assay showed that after 48 h of incubation, there was a slight but not significant decrease in number of cells transfected with si-EZH2 or treated with GSK126 compared with respective control cells (data not shown). The number of invading cells was quantified by Giemsa staining. Relative invasion activities are expressed as the fold-change over their respective controls.

### Mouse experiment

Fifty virgin female BALB/C mice (7 weeks old with average weight of 18–20 g), were purchased from Shanghai BiKai Laboratory Animal Center (Shanghai, China). Mice were maintained and housed on a 12:12 light-dark cycle with access to food and water ad libitum. All experiments were performed under the “Guide for Care and Use of Laboratory Animals” (National Research Council, 1996, National Academy Press, Washington, DC), and approved by the institutional Ethics review board of Shanghai OB/GYN Hospital, Fudan University.

After 1 week of acclimatization, 12 were randomly selected from the 50 mice as donors from whom endometrial tissue fragments were harvested, and another 24 were randomly designated as recipients. The remaining 14 mice received a sham induction. Prior to the endometriosis-inducing and sham induction procedures (as shown below), all mice except donor mice were subjected to a baseline hotplate test^56^ and weighted (day 0). Two weeks after surgery (day 14), all mice were again administrated the hotplate test and weighed, and the mice underwent a procedure to induce endometriosis were randomly divided into 3 groups of equal sizes: Untreated group (U, n = 8), low-dose DZNep group (L, n = 8) and high-dose DZNep group (H, n = 8). The mice underwent a sham induction were randomly divided into 2 groups of equal sizes: blank control group (B, n = 7) and specificity-control group (S, n = 7). The mice in group L received intra-peritoneal (i.p.) injection of DZNep (1 mg/kg bodyweight) dissolved in 0.2 ml sodium sulfate solution every other day for 9 days, while mice in group H and S received i.p. injection of DZNep of 2.5 mg/kg with the same solvent, volume and schedule as that of group L. Mice in groups U and B received i.p. injection of just solvent of the same volume with the identical schedule as that of groups L, H, and S. All groups received identical care.

On day 23 (9 days after the start of i.p. treatment), all mice were subjected to a final hotplate test and weighed and sacrificed. The endometriotic lesions and uterus were retrieved for histological assessment and immunohistochemistry evaluation. The extent of endometriosis was evaluated by assessing the dry weight of lesions.

### Endometriosis induction procedure

The procedure has been described previously^36, 53^. Briefly, after 1 week of acclimatization, the donor mice initially received subcutaneous injection of 0.2 mg/kg 17β-estradiol solution twice a week for a week. Then the donors were sacrificed, and their uteri were removed, and seeded in a Petri dish containing warmed saline and split longitudinally with a pair of scissors. Each uterine horn was minced with scissors and cut into tissue pieces with a maximal diameter consistently smaller than 1 mm. The endometrial fragments were then injected i.p. into mice in group U, L, and H. Each mouse received endometrial tissue fragments derived from a single uterine horn. Mice receiving a sham induction were injected i.p. with fat tissue fragments of similar sizes to that of the uterine tissues.

### Preparation of platelets

Preparation of platelets was performed as described previously^7^. Briefly, 20 ml of peripheral blood samples, donated by male healthy volunteers who had not used any drug at least 3 months prior to the donation, were obtained from the median cubital vein and mixed with 3.2% citric acid serving as an anticoagulant. The platelet-rich plasma (PRP) was obtained by centrifugation at 150 g for 10 min. The pellet fraction was separated from the PRP by centrifugation at 1000 g for 10 min. About 2 × 10^7^ platelets were collected from 1 ml of blood. The platelets were diluted with cell culture medium. Finally, a total of ~2 × 10^7^ platelets/ml was added into cell culture dishes. Cells were treated with either buffer (phosphate buffer saline, or PBS), platelets, platelets activated with thrombin 0.5 U/ml (T8885, Sigma-Aldrich, St. Louis, MO, USA), or thrombin alone equivolumetrically for 48 h. Thrombin was supplemented within 15 min after dilution with PBS before deactivation. To remove platelets in protein assays, we washed the cells co-cultured with platelets with sterile PBS thrice, as reported previously^57^.

### Statistical analysis

The data are expressed as mean ± s.d. of three independent experiments, each performed in triplicate. The level of significance was determined using Paired-Student’s *t*-test and Wilcoxon signed rank sum test, respectively. To adjust for possible confounding factors, such as age, parity, and menstrual phase, on immunostaining levels in ectopic, eutopic and control endometrium, multiple linear regression analysis was used, with the source of tissue samples being identified by 2 dummy variables: ectopic endometrium (*x*_*1*_ = 1) or not (*x*_*1*_ = 0), eutopic endometrium (*x*_*2*_ = 1) or not (*x*_*2*_ = 0). The immunostaining levels were either log- or square-root transformed to enhance normality when appropriate. To see whether DZNep treatment and other possible factors were associated with hotplate latency or various IHC markers, multiple linear regression models were also used. *P* values of less than 0.05 were considered statistically significant. All computations were made with R 3.3.2^58^.

## Electronic supplementary material

Supplementary Information
