# Supplementary material for: Enhancer of Zeste homolog 2 (EZH2) induces epithelial-mesenchymal transition in endometriosis
Source: Sci Rep. 2017 Jul 28;7:6804. doi: 10.1038/s41598-017-06920-7 (PMC5533797; doi:10.1038/s41598-017-06920-7)
Supplement: Supplementary file 1 — Supplementary Information [file 41598_2017_6920_MOESM1_ESM.doc]

**Supplementary information**

**Enhancer of Zeste homolog 2 (EZH2) induces epithelial-mesenchymal transition**

**in endometriosis**

Qi Zhang, Peixin Dong, Xishi Liu, Noriaki Sakuragi & Sun-Wei Guo

**Content**

**Table S1**: Body weight (in grams) of the indicated groups of mice.

**Table S2**: Hotplate latency (in seconds) of the indicated groups of mice.

**Table S3**: Antibodies used for western blotting and immunohistochemistry analysis.

**Table S4**: Primers used for real-time RT-PCR analysis.

**Table S5:** The raw data for quantitative Western blot analysis shown in Figure 1D.

**Table S6:** The raw data for quantitative Western blot analysis shown in Figure 5.

**Figure S1**: Representative immunohistochemical staining for EZH2 and EMT markers in ectopic endometriotic lesions of mice treated with vehicle or DZNep.

**Figure S2**: Immunohistochemical analysis of EZH2, EED, SUZ12, H3K27me3 and H3K9me3 expression in eutopic/control endometrium from mice with or without endometriosis induction, and received the treatment with vehicle or DZNep.

**Table S1.** Body weight (in grams) of the indicated groups of mice.

| Group | Before endometriosis induction (mean ± s.d.) | 14 days after endometriosis induction (mean ± s.d.) | 9 days after treatment  (mean ± s.d.) |
| --- | --- | --- | --- |
| U | 22.7 ± 1.0 | 23.4 ± 1.3 | 24.2 ± 1.0 |
| L | 22.7 ± 1.1 | 23.0 ± 1.1 | 24.3 ± 0.8 |
| H | 22.9 ± 1.2 | 23.5 ± 1.4 | 24.0 ± 1.1 |
| B | 21.9 ± 0.6 | 22.3 ± 1.1 | 23.2 ± 1.0 |
| S | 21.8 ± 0.9 | 22.3 ± 1.1 | 23.7 ± 0.8 |

The mice with induced endometriosis were randomly divided into 3 groups: Untreated group (U, n = 8), low-dose DZNep group (L, n = 8) and high-dose DZNep group (H, n = 8). The mice with sham injection were randomly divided into two groups: blank control group (B, n = 7) and specificity control (S, n = 7) group. The body weight of each group was determined at indicated time points.

**Table S2.** Hotplate latency (in seconds) of the indicated groups of mice.

| Group | Before endometriosis induction (mean ± s.d.) | 14 days after endometriosis induction (mean ± s.d.) | 9 days after treatment  (mean ± s.d.) |
| --- | --- | --- | --- |
| U | 21.0 ± 4.2 | 11.3 ± 4.7 | 9.3 ± 1.0 |
| L | 21.0 ± 3.8 | 13.1 ± 3.7 | 14.1 ± 0.8 |
| H | 21.5 ± 4.4 | 12.4 ± 4.6 | 18.5 ± 1.1 |
| B | 18.9 ± 2.5 | 18.1 ± 3.3 | 17.1 ± 1.0 |
| S | 20.1 ± 4.5 | 18.4 ± 3.8 | 18.7 ± 0.8 |

The mice with induced endometriosis were randomly divided into 3 groups: Untreated group (U, n = 8), low-dose DZNep group (L, n = 8) and high-dose DZNep group (H, n = 8). The mice with sham injection were randomly divided into two groups: blank control group (B, n = 7) and specificity control (S, n = 7) group. The hotplate latency of each group was determined at indicated time points.

**Table S3.** Antibodies used for western blotting and immunohistochemistry analysis.

| Protein | Manufacturer | Dilution (western blotting/immunohistochemistry) |
| --- | --- | --- |
| EZH2 | Cell Signaling | 1:1000/1:50 |
| EED | Abcam | 1:1000/1:50 |
| SUZ12 | Abcam | 1:1000/1:50 |
| H3K27me3 | CST | 1:1000/1:100 |
| H3K9me3 | Abcam | 1:1000/1:200 |
| E-cadherin | CST | 1:1000/1:400 |
| Vimentin | Abcam | 1:1000/1:100 |
| Alpha smooth muscle actin (α-SMA) | Abcam | —/1:100 |
| Collagen I | Abcam | —/1:100 |
| Slug | Abcam | 1:1000/— |
| GAPDH (loading control) | CST | 1:1000/— |

**Table S4.** Primers used for real-time RT-PCR analysis.

| Gene | Sequence (5'→3') | |
| --- | --- | --- |
| *EZH2* | Forward | 5’-GGACGAAGAATAATCATGGGCC-3’ |
|  | Reverse | 5’-CGTCTGAACCTCTTGAGCTGTCT-3’ |
| *EED* | Forward | 5’-TGGACAAGTAAAGAAGGAGATCC-3’ |
|  | Reverse | 5’-TTGCAACAACCGGATTTCTC-3’ |
| *SUZ12* | Forward | 5’-GATAAAAACAGGCGCTTACAGCTT-3’ |
|  | Reverse | 5’-AGGTCCCTGAGAAAATGTTTCGA-3’ |
| *Vimentin* | Forward | 5’-GAACGCCAGATGCGTGAAATG-3’ |
|  | Reverse | 5’-CCAGAGGGAGTGAATCCAGATTA-3’ |
| *N-cadherin* | Forward | 5’-ATCCTACTGGACGGTTCG-3’ |
|  | Reverse | 5’-TTGGCTAATGGCACTTGA-3’ |
| *Fibronectin* | Forward | 5’-CCATCGCAAACCGCTGCCAT-3’ |
|  | Reverse | 5’- AACACTTCTCAGCTATGGGCTT-3’ |
| *PAI-1* | Forward | 5’-ACCGCAACGTGGTTTTCTCA-3’ |
|  | Reverse | 5’-TTGAATCCCATAGCTGCTTGAAT-3’ |
| *Snail* | Forward | 5’-TCGGAAGCCTAACTACAGCGA-3’ |
|  | Reverse | 5’- AGATGAGCATTGGCAGCGAG-3’ |
| *Slug* | Forward | 5’-AAGCATTTCAACGCCTCCAAA-3’ |
|  | Reverse | 5’-GGATCTCTGGTTGTGGTATGACA-3’ |
| *GAPDH* | Forward | 5’-GCACCGTCAAGGCTGAGAAC-3’ |
|  | Reverse | 5’-TGGTGAAGACGCCAGTGGA-3’ |

**Table S5.** The raw data for quantitative Western blot analysis shown in Figure 1D.

| Group | (mean ± s.d.) | *p-*value |
| --- | --- | --- |
| Normal endometrium | 1.00 ± 0.72 | 0.013 |
| Eutopic endometrium from endometriosis | 2.40 ± 1.27 |

| Group | (mean ± s.d.) | *p-*value |
| --- | --- | --- |
| Normal endometrial epithelial cell | 0.98 ± 0.31 | 0.011 |
| Endometriotic epithelial cell | 1.72 ± 0.45 |

**Table S6.** The raw data for quantitative Western blot analysis shown in Figure 5.

| Protein | Group | (mean ± s.d.) | *p-*values |
| --- | --- | --- | --- |
| EZH2  (n = 3) | Buffer | 1.00 ± 0.16 | Buffer vs. Platelets = 0.044  Buffer vs. Activated platelets = 0.037  Buffer vs. Thrombin > 0.05 |
|  | Platelets | 1.58 ± 0.13 |  |
|  | Activated platelets | 1.54 ± 0.32 |  |
|  | Thrombin alone | 0.83 ± 0.16 |  |
| H3K27me3  (n = 5) | Buffer | 1.00 ± 0.84 | Buffer vs. Platelets = 0.043  Buffer vs. Activated platelets = 0.043  Buffer vs. Thrombin > 0.05 |
|  | Platelets | 1.72 ± 1.37 |  |
|  | Activated platelets | 1.93 ± 1.56 |  |
|  | Thrombin | 0.89 ± 0.69 |  |
| H3K9me3  (n = 7) | Buffer | 1.00 ± 0.31 | Buffer vs. Platelets = 0.018  Buffer vs. Activated platelets = 0.018  Buffer vs. Thrombin > 0.05 |
|  | Platelets | 1.40 ± 0.48 |  |
|  | Activated platelets | 2.08 ± 0.85 |  |
|  | Thrombin alone | 0.91 ± 0.32 |  |


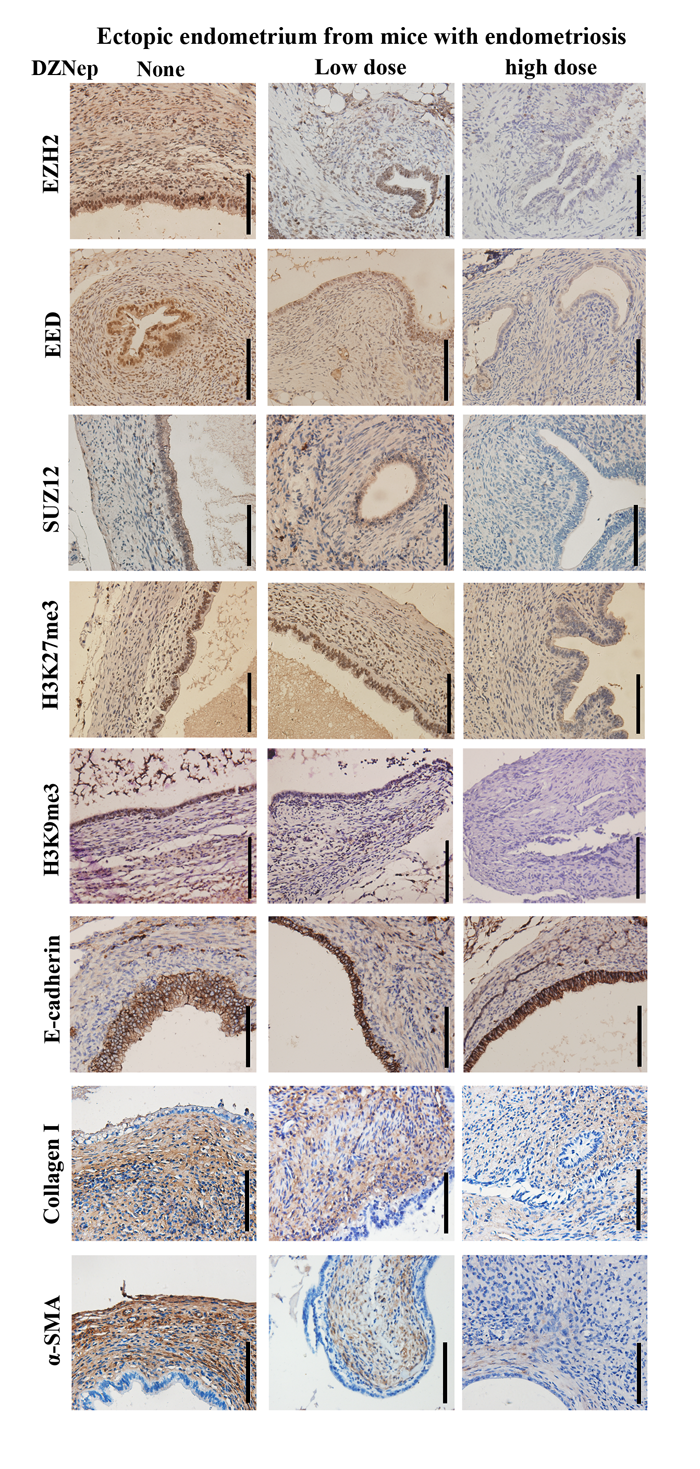


**Figure S1.** **Representative immunohistochemical staining for EZH2 and EMT markers in ectopic endometriotic lesions of mice treated with vehicle or DZNep.**

Note the nuclear expression of EZH2, EED, SUZ12, H3K27me3 and H3K9me3 in both stromal and epithelial cells of ectopic lesions (mostly in epithelial cells). E-cadherin staining was found in cytoplasm and cell membranes of epithelial cells. Collagen I and α-SMA staining was seen mostly in the stromal components of ectopic lesions. Scale bar = 125 µm.


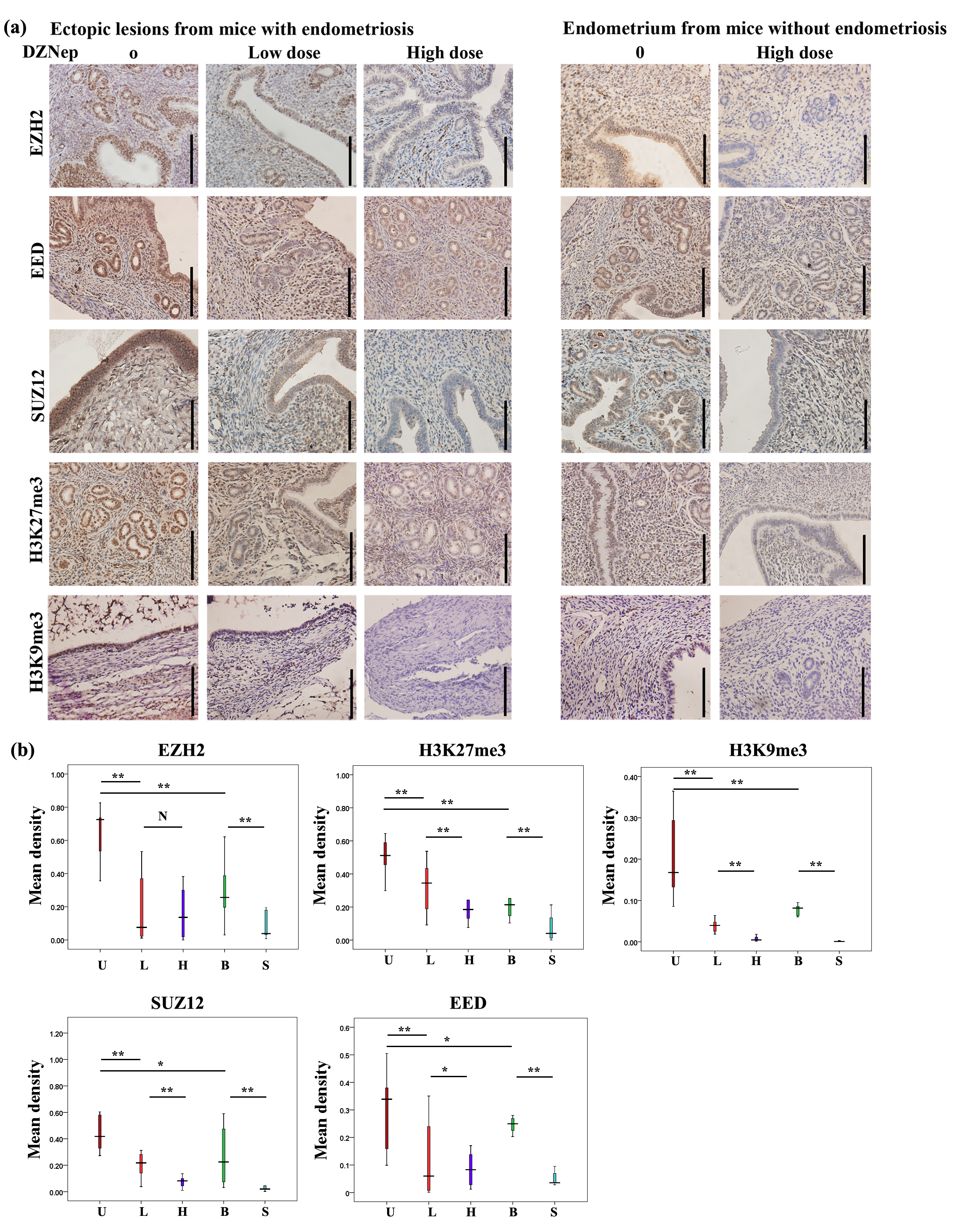


**Figure S2.** **Immunohistochemical analysis of EZH2, EED, SUZ12, H3K27me3 and H3K9me3 expression in eutopic/control endometrium from mice with or without endometriosis induction, and received the treatment with vehicle or DZNep.**

(**a**) Representative immunohistochemistry images of EZH2, EED, SUZ12, H3K27me3 and H3K9me3 in eutopic endometrium of mice with or without endometriosis induction and received the treatment with vehicle or DZNep. The mice with induced endometriosis were randomly divided into 3 groups of equal sizes. Untreated group (U, n = 8), low-dose DZNep group (L, n = 8) and high-dose DZNep group (H, n = 8). The mice with sham injection were randomly divided into two groups: blank control group (B, n = 7) and specificity control group (S, n = 7). Note the nuclear expression of EZH2, EED, SUZ12, H3K27me3 and H3K9me3 in both stromal and epithelial cells of ectopic lesions (mostly in epithelial cells). Scale bar = 125 μm. (**b**) Box plot shows the data of the quantitative immunohistochemical expression of EZH2, EED, SUZ12, H3K27me3, H3K9me3 in epithelial cells of indicated groups. *: *P* < 0.05; **: *P* < 0.01; N: not statistically significant.
